# Exploring the association between smoking and serum magnesium in type 2 diabetes: A cross-sectional retrospective study

**DOI:** 10.1097/MD.0000000000046172

**Published:** 2025-11-28

**Authors:** Müslüm Güneş

**Affiliations:** a Department of Internal Medicine, Gazi Yaşargil Hospital, Diyarbakir, Turkey.

**Keywords:** cross-sectional study, diabetes mellitus, glucose, magnesium, smoking

## Abstract

Magnesium has numerous important functions in human metabolism. Low magnesium level is a common finding in type 2 diabetes mellitus (T2DM) but the causes have not been sufficiently investigated. Smoking is associated with major health problem but the link between magnesium and smoking has been little investigated. This study aimed to compare smokers and nonsmokers in terms of magnesium data, to compare hypomagnesemics and normomagnesemics in terms of smoking data, and to investigate the correlation between data related to magnesium and smoking in T2DM. In this study, a total of 100 male patients with T2DM were investigated. Smokers (n = 35) and nonsmokers (n = 65) were compared in terms of serum magnesium level and magnesium depletion score. Hypomagnesemics (n = 23) and normomagnesemics (n = 77) were compared in terms of smoking intensity, smoking duration, and smoking pack-year. Correlation analysis was performed between data related to magnesium and smoking in T2DM. Statistical results showed that there was no significant difference between smokers and nonsmokers in terms of serum magnesium level (*P* = .822) and magnesium depletion score (*P* = .522). There was no significant difference between hypomagnesemics and normomagnesemics in terms of smoking intensity (*P* = .918), smoking duration (*P* = .923), and smoking pack-year (*P* = .908). No significant correlation was found between mentioned parameters related to magnesium and smoking data (*P* > .05). The present analysis did not support a significant association between smoking and serum magnesium in T2DM. The literature evaluating the relationship between these 2 conditions is limited. Our findings suggest smoking is not an independent determinant of hypomagnesemia in T2DM, contrary to prior reports in healthy populations.

## 1. Introduction

Type 2 diabetes mellitus (T2DM) is a long-lasting disease associated with high morbidity and mortality due to its symptoms, comorbidities, and various complications.^[1,2]^ Currently, the global prevalence of T2DM has exceeded 400 million^[1]^ and is known to be increasing.^[3]^ The pathophysiology of T2DM is complex and involves increased insulin resistance and dysfunction of β-cells and mitochondria, and oxidative stress.^[1]^ Considering the severity of T2DM, early diagnosis and effective treatment are very important. Management is based on pharmacological and lifestyle interventions such as physical activity and diets, but there is currently no complete remission.^[1,4]^

Smoking is a common habit associated with significant health issues such as malignancies,^[5]^ cardiovascular diseases,^[6]^ and T2DM.^[7]^ This habit may cause illness or worsen a preexisting condition. In addition to its association with clinical problems, smoking has also been shown to be linked to abnormal laboratory findings such as hypomagnesemia.^[8]^ Smoking may affect magnesium levels through oxidative stress, kidney function, or nutritional factors. However, the effects of smoking on clinical and laboratory outcomes are multifaceted and require further research.

Magnesium is a vital mineral that plays important roles in human metabolism as a cofactor or activator of numerous biochemical enzymatic reactions.^[9,10]^ Hypomagnesemia predisposes to various serious diseases such as diabetes mellitus^[11,12]^ and osteoporosis.^[13]^ Despite the high clinical importance of magnesium and its abundant natural supply, underdiagnosis and hypomagnesemia are common.^[11]^ Therefore, it is important to investigate factors that have the potential to determine hypomagnesemia. It has been reported that some conditions including alcoholism and malabsorption and various drugs including proton pump inhibitors, diuretics, and metformin are associated with hypomagnesemia.^[10,14]^ In clinical practice, serum magnesium level is the easiest and most commonly used method to assess magnesium status in the body.^[9]^ However, this method has limited power in determining the total magnesium in the body. Nevertheless, there is a positive correlation between ionized and total magnesium levels.^[12]^ In recent studies, the magnesium depletion score has been found useful as an indicator of total magnesium status and is frequently used for this purpose.^[15]^

Although the development of T2DM is not fully understood, some associated factors such as hypomagnesemia^[12]^ and smoking^[16]^ have been identified. Although hypomagnesemia is well known to be associated with T2DM,^[12]^ the underlying mechanisms of this relationship have also not been fully elucidated. Given that both smoking and hypomagnesemia are associated with T2DM,^[12,16]^ there may hypothetically be a relationship between these 2 conditions in T2DM patients. However, the link between magnesium and smoking has been little investigated in healthy subjects.^[8]^ Furthermore, only 1 study has investigated the link between smoking and magnesium in T2DM patients.^[17]^ Theoretically, smoking and magnesium may be interrelated in T2DM and it is worth investigating whether smoking is linked to hypomagnesemia in T2DM patients. In this context, the aim of this study was to evaluate the relationship between smoking and magnesium in T2DM patients.

## 2. Materials and methods

After obtaining ethical approval (number: 421; date: April 11, 2025), the data of patients with T2DM admitted to our outpatient clinic between December 2024 and April 2025 were analyzed retrospectively. Due to the retrospective design of the study, informed consent was not obtained from the patients. The study was conducted in accordance with the ethical standards of the Declaration of Helsinki.

The inclusion criteria determined and applied for the study were as follows: Individuals with T2DM, age > 18 years, using oral antidiabetics and having the necessary data for this study. The exclusion criteria determined and applied for the study were as follows: Type 1 diabetes mellitus, geriatric patients (≥65 years), insulin users, missing data required for this study, presence of active infection, magnesium therapy, malignancy, psychosis, severe anemia, and female gender.

In this study, serum magnesium levels and other biochemical parameters were measured with Architect Plus c16000 (Abbott, IL). As magnesium parameters, serum magnesium level and magnesium depletion score were used. As smoking data, intensity (number of cigarettes/day), duration (years), and pack-year scores were used.

In this study, data from a total of 100 male patients with T2DM were analyzed. Due to the very low smoking rate in female patients (8%) and the association of gender with serum magnesium levels,^[18]^ only male patients were included in the study.

Demographic characteristics and laboratory features of the patients were investigated using hospital records. Magnesium depletion score was calculated using components including diuretic use (1 point), proton pump inhibitor use (1 point), heavy alcohol consumption (1 point), and renal function based on eGFR (1 point: 60 mL/min/1.73 m^2^–90 mL/min/1.73 m^2^; 2 point: <60 mL/min/1.73 m^2^).^[19]^

Smokers (n = 35) and nonsmokers (n = 65) were statistically compared in terms of serum magnesium level and magnesium depletion score. Hypomagnesemics (n = 23) and normomagnesemics (n = 77) were statistically compared in terms of smoking intensity and smoking duration. In addition, statistical correlation analyses were performed between magnesium scores (serum magnesium and magnesium depletion score) and smoking data (intensity, duration, and pack-year) in T2DM.

### 2.1. Statistical analysis

Statistical analysis was performed in accordance with the statistical data editing in scientific articles.^[20]^ The SPSS program, version 27, was used for statistical analysis. The Kolmogorov–Smirnov test was used to evaluate whether the continuous variables were normally distributed. The continuous variables were expressed as mean ± SD (min–max) and median (IQR) for independent sample *t* test and Mann–Whitney *U* test, respectively. Categorical variables were presented as frequency (percentage). Fisher Exact test was used for categorical variables. In addition, statistical analysis was applied to evaluate the significance of correlations between magnesium parameters (serum magnesium and magnesium depletion score) and smoking data (intensity, duration, and pack-year) in smokers (n = 35). *P*-value < 0.05 was accepted as statistically significant level.

## 3. Results

**Figure 1.**
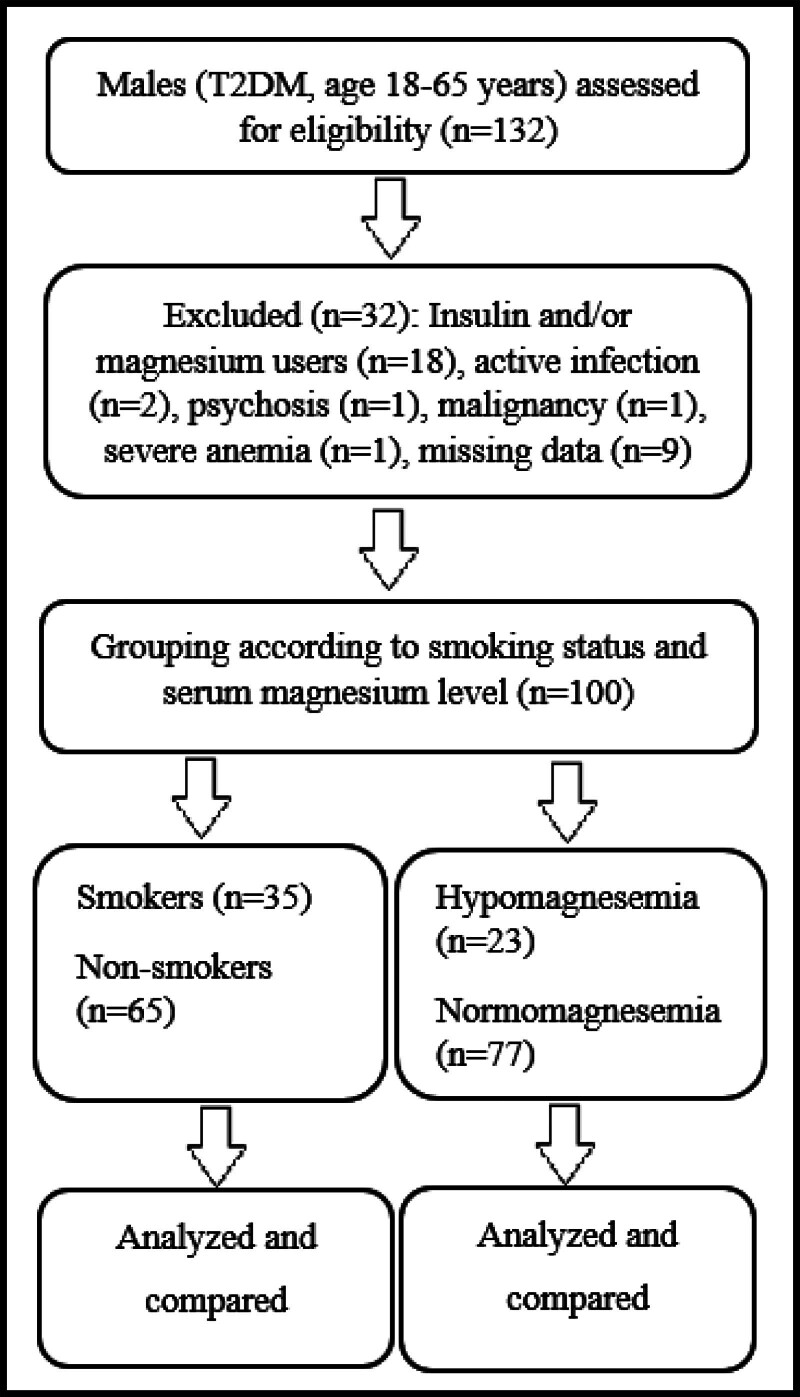
Study flowchart: Male patients with T2DM (n = 132, ages 18–65) were assessed for eligibility. Thirty-two patients were excluded based on the selection criteria. A total of 100 patients were grouped, analyzed, and statistically compared based on smoking status and serum magnesium levels.

The study included 100 males with T2DM, of which 35 (35%) were smokers and 65 (65%) were nonsmokers, 23 (23%) were hypomagnesemics and 77 (77%) were normomagnesemics. Figure 1 schematizes the flow of the study. Demographic characteristics and medication information and laboratory features of the patients are presented in Tables 1 and 2, respectively.

**Table 1:** Demographic characteristics of the patients included in the study.

Characteristics	Data of patients (n = 100)
Age, yr	55.39 ± 7.59 (30–64)
Weight, kg	82.93 ± 12.06 (60–125)
Height, m	1.73 ± 0.06 (1.56–1.90)
Body mass index, kg/m^2^	27.83 ± 3.55 (19.71–35.92)
Diabetes mellitus duration, yr	7.04 ± 5.43 (0.50–25.0)
Hypertension, n (%)	34 (34)
Hyperlipidemia, n (%)	15 (15)
Coronary artery disease, n (%)	14 (14)
Chronic kidney disease, n (%)	13 (14)
Proton pump inhibitor user, n (%)	32 (32)
Metformin user, n (%)	86 (86)
Diuretic user, n (%)	13 (13)
Smoking intensity, n/d	7.25 ± 10.08 (0–35)
Smoking duration, yr	9.65 ± 14.74 (0–50)
Smoking pack-year	10.16 ± 16.27 (0–78.75)

**Table 2:** Laboratory features of the patients included in the study.

Characteristics	Data of patients (n = 100)
Leukocyte, 10^3^/mL	8.28 ± 1.96 (3.83–13.49)
Erythrocyte, 10^3^/mL	5.36 ± 0.48 (4.10–7.27)
Platelet, 10^3^/mL	264.08 ± 56.03 (136.0–415.0)
Neutrophil, 10^3^/mL	4.84 ± 1.51 (2.26–9.65)
Lymphocyte, 10^3^/mL	2.63 ± 0.79 (0.92–4.70)
Monocyte, 10^3^/mL	0.54 ± 0.17 (0.15–1.02)
Creatinine, mg/dL	0.85 ± 0.17 (0.57–1.57)
eGFR, mL/min/1.73 m^2^	100.45 ± 13.62 (49.53–125.10)
Uric acid, mg/dL	4.67 ± 1.17 (2.10–8.10)
Aspartate aminotransferase, U/L	21.15 ± 8.30 (11–70)
Alanin aminotransferaz, U/L	26.73 ± 15.25 (8–99)
Lactate dehydrogenase, U/L	170.32 ± 33.25 (99–281)
Gamma glutamyl transferase, U/L	37.25 ± 32.23 (12–215)
Albumin, g/L	43.77 ± 2.86 (36–51)
Glucose, mg/dL	171.18 ± 73.24 (61–401)
HbA1c	7.83 ± 1.68 (4.60–13.40)
Magnesium, mg/dL	1.92 ± 0.18 (1.49–2.42)
Hypomagnesemia, n (%)	23 (23)
Normomagnesemia, n (%)	77 (77)
Magnesium depletion score	0.0 (1.0)
	0.68 ± 0.92 (0.0-4.0)

eGFR = estimated glomerular filtration rate (2021 CKD-EPI creatinine equation).

In Table 3, statistical comparisons between smokers and nonsmokers in terms of magnesium data are presented. There was no significant difference between smokers and nonsmokers in terms of serum magnesium level (1.93 ± 0.19 vs 1.92 ± 0.17; *P* = .822) and magnesium depletion score (0.74 ± 0.95 vs 0.65 ± 0.91; *P* = .522).

**Table 3:** Comparison between smokers and nonsmokers.

	Smokers (n = 35)	Nonsmokers (n = 65)	*P* (95% CI)
Magnesium, mg/dL	1.93 ± 0.19 (1.49–2.26)	1.92 ± 0.17 (1.63–2.42)	.822*
			(‐0.08 to 0.07)
Hypomagnesemia, n (%)	8 (22.9)	15 (23.1)	1.0
Normomagnesemia, n (%)	27 (77.1)	50 (76.9)	1.0
Magnesium depletion score	0.0 (1.0)	0.0 (1.0)	0.562†
	0.74 ± 0.95 (0.0–4.0)	0.65 ± 0.91 (0.0–4.0)	(0.00–0.00)

Magnesium depletion score was expressed as median (IQR) mean ± SD (min-max).

* Independent sample *t* test.

† Mann–Whitney *U* test.

In Table 4, statistical comparisons between hypomagnesemics and normomagnesemics in terms of smoking data are presented. There was no significant difference between hypomagnesemics and normomagnesemics in terms of smoking intensity (6.96 ± 9.74 vs 7.34 ± 10.25; *P* = .918), smoking duration (9.35 ± 14.41 vs 9.74 ± 14.93; *P* = .923), and pack-year (9.35 ± 14.41 vs 10.41 ± 16.87; *P* = .908).

**Table 4:** Comparison between hypomagnesemia and normomagnesemia.

	Hypomagnesemia (n = 23)	Normomagnesemia (n = 77)	*P* (95% CI)
Smokers, n (%)	8 (34.78)	27 (35.06)	1.0
Nonsmokers, n (%)	15 (65.22)	50 (64.94)	1.0
Smoking intensity, n/d	0.0 (20.0)	0.0 (20.0)	.918*
	6.96 ± 9.74 (0.0–20.0)	7.34 ± 10.25 (0.0–35.0)	(0.00–0.00)
Smoking duration, yr	0.0 (20.0)	0.0 (20.0)	.923*
	9.35 ± 14.41 (0.0–50.0)	9.74 ± 14.93 (0.0–50.0)	(0.00–0.00)
Smoking pack-year	0.0 (20.0)	0.0 (20.0)	.908*
	9.35 ± 14.41 (0.0–50.0)	10.41 ± 16.87 (0.0–78.75.0)	(0.00–0.00)

Smoking data were expressed as median (IQR) mean ± SD (min-max).

* Mann–Whitney *U* test.

In Table 5, statistical correlations between magnesium scores and smoking data are presented. No significant correlation was found between magnesium data (serum magnesium level and magnesium depletion score) and smoking data (smoking intensity, smoking duration, and smoking pack-year) (*P* > .05).

**Table 5:** Correlation of magnesium scores and smoking data the patients.

n = 100	Magnesium, mg/dL	Magnesium depletion score
Smoking intensity, n/d	*r*: 0.049	*r*: 0.061
	*P* = .630	*P* = .546
Smoking duration, yr	*r*: 0.045	*r*: 0.082
	*P* = .654	*P* = .420
Smoking pack-year	*r*: 0.044	*r*: 0.083
	*P* = .662	*P* = .411

Spearman correlation test has been applied.

## 4. Discussion

In this cross-sectional study, we aimed to evaluate the relationship between smoking and magnesium status in patients with T2DM. For this purpose, smokers and nonsmokers were compared in terms of magnesium status, and also hypomagnesemics and normomagnesemics were compared in terms of smoking status. We also evaluated whether there was a significant relationship between smoking status (intensity, duration, and pack-year) and magnesium status (serum level and depletion score). In conclusion, the findings of this study did not support the hypothesis that smoking may be associated with hypomagnesemia status in patients with T2DM. Therefore, smoking may not be a contributing factor to hypomagnesemia, a well-known finding in T2DM.

In the studies conducted on the subject to date, magnesium status in healthy smokers and nonsmokers has been evaluated and compared, and these studies have obtained findings supporting the relationship between smoking and hypomagnesemia.^[8,21–23]^ This relationship has been examined in also T2DM, but in only 1 study.^[17]^ Therefore, the present study is the second study to investigate the relationship between smoking and magnesium in patients with T2DM. Similar to the results of the first study on the subject,^[17]^ our findings show that there is no significant relationship between smoking and hypomagnesemia in patients with T2DM. Considering that both smoking and hypomagnesemia are associated with T2DM^[12,16]^ and that the link between smoking and hypomagnesemia has been demonstrated in healthy individuals,^[8,21–23]^ there was a sensible background to investigate this link in diabetics. Considering that the relevant mechanisms underlying hypomagnesemia in T2DM are not sufficiently clear and there is insufficient literature on the relationship between smoking and hypomagnesemia in T2DM, this topic seems worthy of further investigation. In addition, since the studies on the topic to date have been carried out only on men,^[8,21–23]^ it is important that future studies should be carried out in a way to include women as well.

The findings that smoking and magnesium are associated with each other in healthy individuals but not in diabetics brings to mind some possibilities. For example, it is not clear whether diabetes may mask the effect of smoking on magnesium levels. Furthermore, the potential confounding effects of medications commonly used in diabetic patients, such as metformin, proton pump inhibitors, and diuretics, cannot be ruled out.

On the other hand, the current study has some limitations, particularly regarding the generalizability of the results. For example, only male individuals within a certain age range were included in the study. Another potential limitation is that the study is a retrospective analysis and therefore there is a risk of missing data. This study also includes weaknesses related to the cross-sectional design, which does not have the capacity to reveal a causal relationship. Additionally, the study was conducted with limited time and data, and no sample size calculation or statistical power analysis was performed. The authors acknowledge that there is a potential for type II error, particularly given the small sample size. Finally, a deep and satisfying discussion was not possible due to the paucity of specific literature on the topic of the study.

## 5. Conclusion

In conclusion, the present analysis did not support a significant association between smoking and magnesium in T2DM. Although increased frequency of both smoking and hypomagnesemia in T2DM is well known, the literature evaluating the relationship between these 2 conditions is limited. Our results indicate no clear link between smoking and hypomagnesemia in T2DM, though larger, prospective, multi-gender studies would be helpful.

## Author contributions

**Conceptualization:** Müslüm Güneş.

**Data curation:** Müslüm Güneş.

**Formal analysis:** Müslüm Güneş.

**Investigation:** Müslüm Güneş.

**Methodology:** Müslüm Güneş.

**Project administration:** Müslüm Güneş.

**Software:** Müslüm Güneş.

**Supervision:** Müslüm Güneş.

**Validation:** Müslüm Güneş.

**Writing – original draft:** Müslüm Güneş.
